# Impact of travel by walk and road on testicular hormones, oxidants, traces minerals, and acute phase response biomarkers of dromedary camels

**DOI:** 10.1016/j.heliyon.2021.e06879

**Published:** 2021-05-01

**Authors:** Ragab H. Mohamed, Amal M. Abo El-Maaty, Amal R. Abd El Hameed, Amal H. Ali

**Affiliations:** a Theriogenology Department, Faculty of Medicine, Aswan University, Egypt; b Animal Reproduction and Artificial Insemination Department, Veterinary Research Division, National Research Center, Dokki, Giza, Egypt

**Keywords:** Acute phase response, Antioxidant status, Camel, Testosterone, Transport stress

## Abstract

This study aimed to compare the effect of truck transport and walk travel on testicular hormones, oxidants, antioxidants and acute-phase responses of camels’ walked from Sudan to the Egyptian quarantine and were transported from the quarantine to the slaughterhouses by trucks. Blood samples were collected from walked camels (N ≤ 30) just arrived at the quarantine (Walk), unloaded (N ≤ 12) from the truck (Truck), and control camels (N ≤ 20). Animals were statistically categorized into Walk travel, Truck transport, and Control, then Total travel (Walk + truck transport) was compared to control. Haptoglobin, fibrinogen, superoxide dismutase (SOD), glutathione peroxidase (GPx), nitric oxide (NO), ascorbic acid, glucose, cholesterol, testosterone, estradiol, iron, copper, ALT, AST, alkaline phosphatase (ALP), total proteins, albumin, and creatinine were measured. Results showed that the travel by walk and truck increased haptoglobin (*P* ≤ 0.0001), fibrinogen (*P* < 0.05), ALT (*P* < 0.05), and creatinine (*P* ≤ 0.0001) but decreased NO (*P* ≤ 0.0001), albumin (*P* < 0.05), Ascorbic acid (*P* < 0.05), testosterone (*P* ≤ 0.0001), ALP (*P* < 0.0001), and glucose (*P* ≤ 0.0001). The declined NO (*P* ≤ 0.0001), Ascorbic acid (*P* ≤ 0.0001), iron (P ≤ 0.005), copper (*P* ≤ 0.023), cholesterol (P > 0.05), total proteins (*P* ≤ 0.0001), albumin (*P* ≤ 0.018), globulins (*P* ≤ 0.001), with increased haptoglobin *(P* ≤ 0.0001), AST (*P* ≤ 0.0001), ALP (*P* ≤ 0.0001), and testosterone (*P* ≤ 0.0001) was evident in camels transported by truck compared to walk transport. In conclusion, transport enhanced the acute phase proteins, retarded kidney function, antioxidant status, and energy but truck produced a significant acute-phase response and adversely affected the oxidant-antioxidant balance, destructed proteins kidney, and liver functions than the long travel by walk.

## 1 Introduction

In the past, the old world dromedary camels were known as the desert ship in the Middle East and they used to carry people and goods from country to country. Currently, camel bulls are being used to carry crops and dung, and they are being imported from Sudan. Camels are raised for their meat and milk. Camel races are set for valuable breeds. Sporadic camel breeding still exists in the Sini and western desert of Egypt. Camels used for meat consumption are still imported from Sudan through cutting long journey on foot with frequent stops for water and rest. Transportation has stressful effects on the health and productivity of the animals (Knowles et al., 1999; Minka et al., 2009). There is an increasing public interest and concern for the welfare of livestock during transportation and its either activation of acute-phase response or decreasing the immunological capacity (Mohammadi et al., 2007; Padalino et al., 2017).

Transport has disrupted homeostasis and metabolism, increased the activity of enzymes and hormones, and modified the acute-phase proteins (Lomborg et al., 2008). In dromedary camels, short road transportation by truck transiently increased cortisol and decreased lymphocytes (Emeash et al., 2016) and the increase of cortisol showed a correlation with increasing the distance (El Khasmi et al., 2015). Camels can tolerate dehydration for twenty days to the loss of nearly one-third of their body water content and adapt their body temperature to the environmental temperature to decrease the loss of water, and their kidneys show increased MDA and GSH but decreased SOD whereas dehydration showed no on MDA, GSH, and SOD of the kidney medulla (Ali et al., 2012, 2020). Camels could adapt to four days of food deprivation showing no change of their plasma glucose, cortisol, total proteins, thyroxine levels, and only plasma and urine urea increased while their body temperature decreased (Dahlborn et al., 1992).

The effect of long-distance (1600km/day) shipping and pre-shipping management on acute-phase protein concentrations was investigated in calves (Arthington et al., 2008). Haptoglobin (Hp) has been proposed as a marker of stress in calves (Alsemgeest et al., 1995; Hickey et al., 2003), pigs (Piñeiro et al., 2007), weaned beef calves (Arthington et al., 2003), bulls (Earley et al., 2010, 2011), and camels (Baghshani et al., 2010; El Khasmi et al., 2015). Superoxide dismutase (SOD) activity increases hydrogen peroxide production protection from reactive oxygen would only be given by a simultaneous increase in catalase and glutathione activities (Frei, 1994) in camels (El Khasmi et al., 2015).

External or internal challenges such as infection, inflammation, surgical trauma, stress, and neoplasia increased the synthesis of acute-phase proteins (APP) in the liver (Greunz et al., 2018; Gruys et al., 2005; Murata et al., 2004) by more than 25% which varied greatly among species (Eckersall, 2000; Gruys et al., 2005; Tothova et al., 2014). Acute-phase reaction (APR) is induced by the pro-inflammatory cytokines IL-1, TNF-α, and IL-6 (Gruys et al., 2005; Petersen et al., 2004). Inflammatory stimulus increased the levels of major APPs 10-fold and normalized again within a short period, whereas the minor APPs showed a smaller and more graduated response that lasted longer (Gruys et al., 2005). Fibrinogen, haptoglobin, and copper increase (positive APPs), but albumin, iron, and zinc decrease (negative) following any inflammatory response (Gruys et al., 2005). Hp concentrations increased not only as of the systemic innate immune system response but also in any conditions not associated with inflammation or tissue damage such as physical stress (Casella et al., 2012). The APPs assay may have the potential for monitoring adverse environmental and/or management stressors, thus enabling better assessment of animal welfare during road transportation (Murata 2007; Murata et al., 2004; Piñeiro et al., 2007). Healthy camels can balance between oxidants and antioxidants markers but adverse environmental stress, inflammation, and parasitic infestation disease disturbed this balance (Kataria et al., 2010). Superoxide dismutase (SOD) is considered the first defense against pro-oxidants (Halliwell and Chirico, 1993). Superoxide dismutase (SOD) activity increases hydrogen peroxide production protection from reactive oxygen would only be given by a simultaneous increase in catalase and glutathione activities (Frei, 1994) in camels (El Khasmi et al., 2015).

Though camel and Goats can synthesize Ascorbic acid in their livers from carbohydrates (Gillespie, 1980) but the administration of Ascorbic acid to goats before loading and transportation ameliorated the adverse effects of loading and transportation stress (Minka and Ayo, 2011).

Androgens usually increase during the breeding season in camels (Yagil and Etzi, 1980). The effect of transport on androgens was not yet recorded. Therefore, this study aimed to compare the effects of the traditional long-distance travel of dromedary camels with those transported by truck and not transported controls during their breeding season on acute-phase reactants, androgens, liver enzymes, kidney function, trace minerals, and oxidants and antioxidants biomarkers.

## 2 Materials and methods

This work was approved by the Institutional Animal Use and Care Committee of Faculty of Veterinary Medicine, Aswan University (IAUACC-AU) number 2018- 01. All the camel owners’ had given oral consent to take blood samples.

### 2.1 Animals and managements

This study included camel bulls (N ≤ 30) of 5–6 years old and 350–500kg bodyweight. Camels (N ≤ 30) were transported from Sudan (Darfur and Donkola) to Quarantine of Abo Simpl at Luxor 300km south of Aswan by a walk along 30–40 days with fixed resting stations. Camels were kept in quarantine for at least 15 days until certified clear from infectious diseases. The truck transported twelve camels to the slaughterhouse 300km. A control group of male camels (N ≤ 10) kept on a private farm were sampled and served as the control. This work was approved by the Institutional Animal Care and Use Committee of Faculty of Veterinary Medicine, Aswan University (IACUC-AU) number 2018- 01. Camels were categorized into the control group, traveled by walk (Walk), road transported by truck (Truck).

### 2.2 Blood sampling

All camel owners had given oral consent to collect blood samples. In accordance with the Institutional Animal Care and Use Committees of the National Research Centre and Aswan University, one blood sample was collected from the Jugular vein in plain vacuum tubes from all camels included in the study. Sera were harvested and stored at -20 °C until hormone assaying and biochemical analysis.

### 2.3 The biochemical analyses

Haptoglobin (**HP3222;** *BEN-BIOCHEMICAL ENTERPRISE S.r.l.-via Toselli,* *www.bensrl.it-info@bensrl.it*), and Fibrinogen (Salucea, The Netherlands) kits had the sensitivity limit of 2.9 mg/dL and 4.5 mg/dL, respectively. They have a within-run precision and Run to Run Precisions of 2.1%.

Superoxide dismutase (SOD), glutathione peroxidase (GPx), ascorbic acid, nitric oxide (NO), copper, iron, and zinc were estimated using commercially available kits (Biodiagnostics, Egypt).

Total proteins (TP), albumin (g/dl) ALT, AST (U/L), Alkaline phosphatase (ALP), creatinine, total cholesterol, and glucose were assayed using MG diagnostics (Science and Technology, www.Biostc.net, Egypt). The minimum detectable limit for both TP and albumin was 1 g/dl; ALT was 4U/L; AST was 7U/L; ALP was 1U/100ml; creatinine was 0.31 m/dl; cholesterol was 5 mg/dl, and glucose was 5 mg/dl. The test intra- and inter-precisions for TP was 2.2% and 2.4%; albumin was 2.66% and 3.1%; ALT was 0.73% and 1.3%; AST was 0.40% and 1.5%; ALP was 2.4 and 2.8%; creatinine was 4.45% and 4.58%; total cholesterol was 1.13% and 1.23%, and for glucose was 1.09% and 1.17%.

### 2.4 Statistical analysis

Simple one-way ANOVA (SPSS 20) was used to study the effect of transport by walk (Walk), by the truck (Truck), and control (Cont.). Duncan's Multiple Range Test was performed to differentiate between significant means at P < 0.05. The independent sample t-test was used to compare the camels transported (Walk + Truck) with the control group.

## 3 Results

It could be noticed from Table (1) that the haptoglobin concentrations of total transported camels (truck + walk) increased (*P* < 0.001) compared to control camels. Haptoglobin concentrations increased (*P* < 0.0001) two times (Walk) and three times (Truck). Concentrations of fibrinogen tended (*P* < 0.05) to increase in total transported camels (truck + walk). This increase of fibrinogen in camels traveled by Walk or transported by Truck showed no significant difference compared to controls. The decreased SOD activity and the increased GPx activity in total transported camels (truck + walk) are not significant. NO concentrations declined (*P* ≤ 0.0001) in total traveled camels and this marked decrease (*P* ≤ 0.0001) is obvious in those camels transported by trucks (Table 1).

**Table 1:** Mean ± standard deviation of haptoglobin, fibrinogen, superoxide dismutase (SOD), glutathione peroxidase (GPx), and nitric oxide (NO) in camels traveled by Walk, transported by Truck, and Control.

Parameters	Total travel	Control	Walk	Truck	P-value
Number	42	10	30	12	
Haptoglobin (mg/dl)	12.79∗∗ ±7.59	5.15^a^ ±4.54	12.20^b^ ±7.81	16.31^c^ ±4.97	0.0001
Fibrinogen (mg/dl)	296.1∗ ±310.8	261.7^ab^ ±119.9	309.4^a^ ±148.8	321.6^b^ ±17.2	NS
SOD U/ml	205.1 ±68.4	218.75 ±44.8	203.13 ±69.2	234.38 ±51.3	NS
GPx (mU/mL)	68.65 ±166.58	37.32 ±25.33	74.67 ±175.34	18.08 ±6.36	NS
NO μmol/L	47.91∗∗ ±32.84	75.23^c^ ±56.23	50.62^b^ ±33.96	26.88^a^ ±2.89	0.0001

Means with different superscripts (a,b,c) are significantly different at P < 0.05, ∗ means significant at P < 0.05, ∗∗means significant at P < 0.0001, non significant (NS).

The concentrations of ascorbic acid declined (*P* < 0.05) in total transported camels (truck + walk) camels and this marked decrease (*P* ≤ 0.0001) is progressive in those camels transported by trucks (Table 2). The concentrations of glucose of all transported camels decreased (*P* ≤ 0.0001) compared to controls. Camels traveled by walk and transported by truck had lower (*P* ≤ 0.0001) glucose than controls. The increased iron concentrations in total traveled camels are not statistically significant, but the walking travel increased (*P* ≤ 0.005) iron concentrations compared to either controls or truck transport. The copper concentrations insignificantly declined in total traveled camels. Truck transport decreased (*P* ≤ 0.023) copper concentrations compared to those traveled by walk and control (Table 2).

**Table 2:** Mean ± standard deviation of Ascorbic acid, glucose, iron and copper in camels traveled by walk, by truck, and control.

Parameters	Total travel	Control	Walk	Truck	P-value
Ascorbic mg/L	140.76∗ ±57.13	171.05^b^ ±90.79	151.77^b^ ±51.16	55.45^a^ ±1.07	0.0001
Glucose mg/dl	79.22∗∗ ±10.03	90.76^b^ ±15.79	78.66^a^ ±10.19	83.25^a^ ±8.07	0.0001
Iron μg/dl	758.44 ±429.52	551.62^a^ ±296.41	776.28^b^ ±429.68	568.16^a^ ±401.63	0.005
Copper μg/dl	558.40 ±345.71	581.54^b^ ±317.78	586.08^b^ ±358.45	263.07^a^ ±50.15	0.023

Means with different superscripts (a,b) are significantly different at P < 0.05, ∗ means significant at P < 0.05, ∗∗ means significant at P < 0.0001.

The total cholesterol concentrations tended (*P* ≤ 0.061) to decrease in camels transported by truck in contrast to the others. The testosterone concentrations were lower in total traveled camels (*P* ≤ 0.0001) especially those traveled by walk than those transported by truck and controls (Table 3).

**Table 3:** Mean ± standard deviation of total cholesterol, testosterone and estradiol concentrations in camels traveled by walk, by truck, and control.

Parameters	Total travel	Control	Walk	Truck	P-value
Cholesterol mg/dl	66.52 ±9.61	66.97^b^ ±20.99	67.76^b^ ±9.53	57.58^a^ ±43.69	0.061
Testosterone ng/ml	1.70∗∗ ±1.23	3.10^b^ ±1.08	1.43^a^ ±0.86	3.84^c^ ±1.59	0.0001
E2 pg/ml	86.86 ±49.05	71.26^a^ ±55.93	84.81^ab^ ±49.05	102.75^b^ ±48.09	NS

Means with different superscripts (a,b,c) are significantly different at P < 0.05, ∗∗ means significant at P < 0.0001, Non significant (NS).

The AST activity increased (*P* ≤ 0.0001) in camels transported by truck compared to controls and those traveled by walk. (*P* ≤ 0.0001) ALT activity increased (*P* < 0.05) in total traveled camels and this increase (*P* ≤ 0.0001) was significant in either camels traveled by walk or transported by truck. ALP concentrations descended (*P* < 0.05) in total traveled camels (Walk + Truck) and both walk and truck transported camels had lower (*P* ≤ 0.0001) ALP than control camels (Table 4).

**Table 4:** Mean ± standard deviation of GOT, GPT and alkaline phosphatase (ALP) concentrations in camels traveled by walk, by truck, and control.

Parameters	Total travel	Control	Walk	Truck	P-value
AST U/L	102.82 ±11.83	106.37^b^ ±14.13	101.06^a^ ±10.60	118.65^b^ ±10.95	0.0001
ALT U/L	37.35∗ ±3.10	30.72^a^ ±4.28	37.50^b^ ±3.21	36.21^b^ ±1.96	0.0001
ALP U/L	52.96∗ ±10.67	75.83^c^ ±11.04	52.00^a^ ±10.65	59.43^b^ ±8.69	0.0001

Means with different superscripts (a,b,c) are significantly different at P < 0.05, ∗ means significant at P < 0.0001.

The concentrations of TP decreased (*P* ≤ 0.0001) for truck transport. Total traveled camels (Walk + Truck) had lower albumin (*P* < 0.05) than control. Either truck or walk travel decreased (*P* ≤ 0.018) albumin, but the truck transport showed statistical significance. Total traveled camels (Walk + Truck) had higher globulins (*P* < 0.05) but those transported by truck had lower (*P* ≤ 0.002) globulins than control and those travel by walk. Creatinine (*P* ≤ 0.0001) ascended in total transported camels. Both truck and walk increased (*P* ≤ 0.0001) creatinine (Table 5).

**Table 5:** Mean ± standard deviation of total proteins, albumin, globulin and creatinine concentrations in camels traveled by walk, by truck, and control.

Parameters	Total travel	Control	Walk	Truck	P-value
T. proteins g/dl	5.58 ±1.03	5.56^b^ ±1.02	5.74^b^ ±0.94	4.48^a^ ±1.00	0.0001
Albumin g/dl	4.72∗ ±1.09	5.17^b^ ±1.03	4.79^ab^ ±1.11	4.22^a^ ±0.90	0.018
Globulins g/dl	1.84∗ ±0.91	1.33^a^ ±0.91	1.96^b^ ±0.89	0.95^a^ ±0.48	0.001
Albumin/globulin	3.68∗∗ ±3.39	9.17 ±14.52	3.38 ±3.26	5.82 ±3.79	0.014
Creatinine mg/dl	2.03∗∗∗ ±0.78	1.42^a^ ±0.30	2.01^b^ ±0.83	2.20^b^ ±0.29	0.0001

Means with different superscripts (a,b,c) are significantly different at P < 0.05. total (T.), ∗ significant at P < 0.05, ∗∗ significant at P < 0.01, ∗∗∗significant at P < 0.0001.

## 4 Discussion

In dromedary camels, haptoglobin (Hp) and fibrinogen showed prolonged response because of their long half-life (Greunz et al., 2018). Similar to the increase of Hp two folds in traveled camels (and three folds in those transported by truck, Hp increased up to Day 11 in transported bulls (Earley et al., 2011), for 4 days in road transported young bulls (Cafazzo et al., 2012), up to two days in transported cattle (Lomborg et al., 2008), Feedlot calves (Murata and Miyamoto 1993), and recently weaned beef calves (Marti et al., 2017) indicating a long-term food deprivation activated the sympathetic nervous system during road transport (Earley et al., 2011). Hp concentrations were greater in road transported feeder cattle (Cooke et al., 2013; Marques et al., 2012), and Holstein heifers (Kang et al., 2017). In contrast to the increase of Hp in traveled camels by walk or transported by truck of this study, Hp did not vary in either male or female camels transported by truck from 1 h before loading up to 24h after unloading with no significant difference between the sexes at any sampling time (Baghshani et al., 2010).

Fibrinogen in dromedary camels is considered a positive minor APP because of its prolonged elevation, for more than 7 days post-intervention (Greunz et al., 2018). In the current study, fibrinogen recorded in truck transported and walk traveled was higher than control, but truck transport increased fibrinogen than walk. In contrast to the increase of fibrinogen in transported camels by the truck of this study, long-distance transported male or female camels showed no increase in fibrinogen up to 24h after unloading (Baghshani et al., 2010) and decreased it following road transportation in calves and bulls (Earley and O'Riordan, 2006; Mohammadi et al., 2007; Marti et al., 2017).

Truck transport induced imbalance in oxidant-antioxidant status, which could be noticed by the insignificant increase of SOD with the decrease of GPx. Similarly, the transport stress expressed by increased activities of SOD with decreased GPx was noticed in camels transported by trucks for a distance >350–360km (El Khasmi et al., 2015). Similar to the non-significant increase of GPx in camels travel by walk, male camels transported during hot summer showed slight insignificant increased GPx activity up to 24h after arrival but the insignificant decrease of GPx in transported by truck was observed in females (Nazifi et al., 2009). This difference between walk and truck transport in the enzymatic antioxidant status indicates that camels traveled by walk had enough time to adapt to this long persistence stress and can delay the imbalance between antioxidant enzymes-oxidants until the non-enzymatic antioxidants (ascorbic acid) became unable to keep this balance resulted from either abrupt (truck transport) or long (Walk) stress.

The decreased concentrations of ascorbic acid in Total travel camels transported show tissue damage and the use of ascorbic acid to balance the produced oxidants. Glucose concentrations in transported and traveled camels of this study were lower than control. Though the values of glucose in our camel are within the normal range (56–158 mg/dl) of young female camels bred for racing (Eltahir et al., 2016), the decrease of glucose in transported camels of our study could be attributed to the decreased food intake, dehydration, and thirst (Kataria and Kataria 2004; Kataria et al., 2007) with the ability of camels to decrease their blood glucose under stressful conditions (Badakhshan and Mirmahmoudi, 2016). In agreement with our results, young bulls got low plasma glucose after transport (Cafazzo et al., 2012). Contrary, the glucose of young camels (3–4 years old) and weighing about 300 kg (Saeb et al., 2010) and Charolais bulls (Earley et al., 2010) was high at the end of transport and returned to basal values 24h after transport termination. Also, camels (El Khasmi et al., 2015), fattened cattle (Malena et al., 2006), horses (Stull and Rodiek, 2000), goats (Minka et al., 2009), and pigs (De Silva and Kalubowila, 2012) transported by truck had gradually increased glucose with the increase of the transport distance that was referred to the restriction of feed intake several hours before loading (Broom et al., 1996), and the resumption of these physiological variables rapidly once received feeding and resting in sheep (Knowles et al., 1995; Knowles and Warriss, 2007). These differences were referred to different handling methods, the differences of trucks, drivers, days travel, and road topographies (El Khasmi et al., 2015), besides breed differences (Browning and Leite-Browning, 2013).

Testosterone is one of the anabolic steroids and the decrease of its concentrations after long walk travel refers to the adaptation of camel to the longer intervals of food and water deprivation by decreasing their metabolic rate (Baghshani et al., 2010). Though total cholesterol of camel transported by truck was lower than long walk travel and control, their cholesterol concentrations lied within the range (40–77 mg/dl) reported for dromedaries (Eltahir et al., 2016; Mohammed et al., 2007). Contrary, camels transported by truck for 5h along 300km during August had a slight non-significant change of 1h during the journey compared up to 5h after transport (Saeb et al., 2010). The decrease of testosterone associated with high cholesterol in camels traveled by walk may result from the lipolysis of the hump fat to keep their cholesterol within normal range after >30–40 days walk through the desert. Similar to the insignificant effect of road transport on testosterone concentrations of the current study, it did not influence testosterone release in stallions (Deichsel et al., 2015).

The increases in liver enzymes indicate liver damage. All transported camels had higher ALT concentrations. The values reported for transported males are similar to the maximum values reported for young not transported female camels (Eltahir et al., 2016). In female dromedaries, AST and ALT enzymes were neither affected by season nor age (Ali et al., 2008). Similar to the increased AST when camels were transported by truck compared to walk travel, horses transported for long distances by truck >20h had increased AST after unloading (Padalino et al., 2017). Camel bulls transported by truck had higher ALP than those traveled long distant by walk, and control camels had the highest ALP concentrations. All camels had ALP concentrations within the normal range (50–187U/L) reported for female young camels (Eltahir et al., 2016).

Not only the stress induced by truck transport decreased iron in camels of this study but also surgical castration and vaccination induced the same effect, but resulted in a more intensive systemic inflammatory stimulus (Greunz et al., 2018). The lower iron concentrations in camel bulls of this study are 5 times higher than the iron levels reported (56-158ug/dl) for young females (Eltahir et al., 2016). This may refer to the difference between gender, breed, and diet. Though copper increases in response to stress and is one of the positive acute-phase responses (Gruys et al., 1999) its decrease in dromedaries transported by trucks reflects the restriction of food. The lowest copper observed in camels transported by truck exceeded the range (54-89ug/dl) previously reported for dromedaries (Baghshani et al., 2010; Eltahir et al., 2016).

In agreement with the decreased total proteins in camel bulls transported by truck, young bulls transported by truck had low total proteins (Padalino et al., 2017) that resulted also after dehydration for 24 days in dromedary camels (Kataria et al., 2007). In contrast, protein concentrations increased from 24h up to day 11 following transport in all transported bulls (Earley et al., 2010, 2011), in horses (Cywinska et al., 2012), in transported male or female camels at unloading (Baghshani et al., 2010), for <24h in transported female camels during the breeding season (Saeb et al., 2010; Tharwat et al., 2013), and in dromedaries transported by walk and truck since the arrival to 18 h later (Emeash et al., 2016) that reflex the long-term food deprivation and transient dehydration (Niedźwiedź et al., 2012). This conflict in the total proteins between our study and transported camels of other studies could be attributed to their adaptation to the adverse transport and environmental conditions.

All camel bulls of this study except control had low albumin which simulates the decrease of albumin in female camels rather than male camels transported by truck (Baghshani et al., 2010), female camels within 2h after unloading (Tharwat et al., 2013), upon arrival up to 4 days in young bulls (Cafazzo et al., 2012) and contradicts the increase of albumin in camels transported by walk or truck till 18 h after arrival (Emeash et al., 2016), road transported young calves (Grigor et al., 2001) and bulls (Earley et al., 2010). This low albumin as one of the negative acute-phase proteins indicated more stress when using trucks due to loading and unloading that overcame the biochemical adaptive patterns of camels to withstand several physiological and pathological stressors (Baghshani et al., 2010) and that the restriction of feed and water was not enough for camels to resume their normal physiological status within 24h. In dromedaries, albumin either did not change (Saeb et al., 2010) or male camels got higher values than females this difference referred to the change of the breed, age, or gender (Eltahir et al., 2016).

All camels except control had a lower albumin/globulin ratio that indicated not only stress but also disturbed immunological status. The decrease of globulins observed here was also observed in transported young bulls at arrival (Padalino et al., 2017). In contrast, globulins increased in camels transported by walk or truck till 18 h after arrival (Emeash et al., 2016) and increased in camels starting from 2h after unloading (Tharwat et al., 2013) and in bulls post-transport (Earley et al., 2010).

All transported camels of this study had higher creatinine compared to control indicating the stress of transport and the dehydration. Though dehydration in winter and summer in dromedary camels decreased creatinine, rehydration increased these levels (Kataria et al., 2007). Values of creatinine of camel bulls of the current study lie within the range of young female camels 1.3–2.2 mg/dl (Eltahir et al., 2016).

## 5 Conclusions

Transport by trucks induced more stress than long-distance walk travel. Camels transported by trucks could be supplemented by ascorbic acid and minerals to minimize the stressful effects of handling then get rest and hydrated as soon as unloaded. The manipulation of camels during loading and unloading has a more stressful impact compared to the travel by walk for very long transport distance so; the maximum care should be taken during this process. Future work should focus on the effect of transport time on the stress oxidant in camel meat.

## Declarations

### Author contribution statement

Ragab H. Mohamed: Conceived and designed the experiments; Performed the experiments.

Amal M. Abo El-Maaty: Analyzed and interpreted the data; Contributed reagents, materials, analysis tools or data; Wrote the paper.

Amal R. Abdelhammed, Amal H. Ali: Contributed reagents, materials, analysis tools or data.

### Funding statement

This research did not receive any specific grant from funding agencies in the public, commercial, or not-for-profit sectors.

### Data availability statement

Data will be made available on request.

### Declaration of interests statement

The authors declare no conflict of interest.

### Additional information

No additional information is available for this paper.
